# Supplementary material for: Does knowledge of the primary tumour affect survival after surgery for spinal metastatic disease? A retrospective longitudinal cohort study
Source: BMJ Open. 2021 Aug 24;11(8):e050538. doi: 10.1136/bmjopen-2021-050538 (PMC8388281; doi:10.1136/bmjopen-2021-050538)
Supplement: Author's manuscript [file bmjopen-2021-050538.draft_revisions.pdf]

BMJ Open is committed to open peer review. As part of this commitment we make the peer review history of every article we publish publicly available.

When an article is published we post the peer reviewers' comments and the authors' responses online. We also post the versions of the paper that were used during peer review. These are the versions that the peer review comments apply to.

The versions of the paper that follow are the versions that were submitted during the peer review process. They are not the versions of record or the final published versions. They should not be cited or distributed as the published version of this manuscript.

BMJ Open is an open access journal and the full, final, typeset and author-corrected version of record of the manuscript is available on our site with no access controls, subscription charges or pay-per-view fees (http://bmjopen.bmj.com).

If you have any questions on BMJ Open's open peer review process please email info.bmjopen@bmj.com

BMJ Open

Does knowledge of the primary tumour affect survival after surgery for spinal metastatic disease? A retrospective longitudinal cohort study

Journal:	*BMJ Open*
Manuscript ID	bmjopen-2021-050538
Article Type:	Original research
Date Submitted by the Author:	22-Feb-2021
Complete List of Authors:	Carrwik, Christian; Uppsala University, Department of Surgical Sciences<br>Olerud, Claes; Uppsala University, Department of Surgical Sciences<br>Robinson, Yohan; Uppsala University, Department of Surgical Sciences; Armed Forces Centre for Defence Medicine, Department of Research and Development
Keywords:	Spine < ORTHOPAEDIC & TRAUMA SURGERY, Neurosurgery < SURGERY, Orthopaedic oncology < ORTHOPAEDIC & TRAUMA SURGERY

SCHOLARONE™
Manuscripts

I, the Submitting Author has the right to grant and does grant on behalf of all authors of the Work (as defined in the below author licence), an exclusive licence and/or a non-exclusive licence for contributions from authors who are: i) UK Crown employees; ii) where BMJ has agreed a CC-BY licence shall apply, and/or iii) in accordance with the terms applicable for US Federal Government officers or employees acting as part of their official duties; on a worldwide, perpetual, irrevocable, royalty-free basis to BMJ Publishing Group Ltd ("BMJ") its licensees and where the relevant Journal is co-owned by BMJ to the co-owners of the Journal, to publish the Work in this journal and any other BMJ products and to exploit all rights, as set out in our licence.

The Submitting Author accepts and understands that any supply made under these terms is made by BMJ to the Submitting Author unless you are acting as an employee on behalf of your employer or a postgraduate student of an affiliated institution which is paying any applicable article publishing charge ("APC") for Open Access articles. Where the Submitting Author wishes to make the Work available on an Open Access basis (and intends to pay the relevant APC), the terms of reuse of such Open Access shall be governed by a Creative Commons licence – details of these licences and which Creative Commons licence will apply to this Work are set out in our licence referred to above.

Other than as permitted in any relevant BMJ Author's Self Archiving Policies, I confirm this Work has not been accepted for publication elsewhere, is not being considered for publication elsewhere and does not duplicate material already published. I confirm all authors consent to publication of this Work and authorise the granting of this licence.

For peer review only

Title

Does knowledge of the primary tumour affect survival after surgery for spinal metastatic disease? A retrospective longitudinal cohort study

Authors

Christian Carrwik MD (corresponding author), Claes Olerud MD PhD, Yohan Robinson MD PhD MBA

Christian Carrwik MD (corresponding author)
christian.carrwik@surgsci.uu.se
Department of Surgical Sciences
Uppsala University
SE-751 85 Uppsala
Sweden
Phone +46-(0)18-611 00 00

Claes Olerud MD PhD
claes.olerud@surgsci.uu.se
Department of Surgical Sciences
Uppsala University

Yohan Robinson MD PhD MBA
yohan.robinson@gu.se

For peer review only
For peer review only - http://bmjopen.bmj.com/site/about/guidelines.xhtml

Does knowledge of the primary tumour affect survival…

Department of Surgical Sciences
Uppsala University
Sweden
Department of Research and Development
Armed Forces Centre for Defence Medicine
Gothenburg
Sweden
Institute of Clinical Sciences
Sahlgrenska Academy, Gothenburg University
Gothenburg
Sweden

Abstract

Objectives: To compare survival after surgery for patients with spinal metastatic disease with known primary tumour (KPT) versus patients with unknown primary tumour (UPT).

Participants: 393 patients 18 years or older (270 men, 123 women, mean age 67.3 years) undergoing surgery at Uppsala University Hospital in Sweden due to spinal metastatic disease 2006-2016. 271 patients (69%) had a KPT at the time of surgery and 122 (31%) had an UPT.

Interventions: Decompressive and/or stabilizing spine surgery due to spinal metastatic disease.

Primary outcome: Survival (median and mean) after surgery.

Results: The estimated median survival time after surgery for patients with KPT was 7.4 months (CI 6.0-8.7) and mean survival time 21.6 months (CI 17.2-26.0). For patients with UPT, the median estimated survival time after surgery was 15.6 months (CI 7.5-23.7) and the

For peer review only - http://bmjopen.bmj.com/site/about/guidelines.xhtml

mean survival time 48.1 months (CI 37.3-59.0). The difference in survival between the KPT and UPT groups was statistically significant (p=0,001)

Conclusion: In this study, patients with spinal metastasis and UPT had a longer expected survival after surgery compared to patients with KPT. This suggests that patients with UPT and spinal metastasis should not be withheld from surgery only based on the fact that the primary tumour is unknown.

Key words: Tumour, spine, metastases, cancer, surgery, survival

Word count: 2142 words.

Strengths and limitations of this study

- Large single-center cohort with at least four years follow-up time
- Reliable survival data with linkage to the Swedish Population Register
- Includes results of biopsies of the unknown primary tumours
- No control group with patients treated only non-surgically
- Lacks reliable data on quality of life and neurologic outcome after surgery

Introduction

The evolution of oncological treatment and longer expected survival has lowered the threshold for surgical intervention among patients with metastatic spine disease in recent decades. In several studies, decompressive surgery (often in combination with surgical stabilization) has been found to be beneficial for patients with an expected survival of at least

For peer review only - http://bmjopen.bmj.com/site/about/guidelines.xhtml

three months.[1 2] In a recent study by Dea et al, this time frame has been challenged and the authors suggest that the extent of the surgery and patient specific factors rather than expected survival should be guiding in the decision-making process.[3]

The prognosis for the individual patient depends on several factors, including the patient's performance status and the extent of generalization of the malignancy. One of the most important factors is the type of primary tumour and the underlying prognosis for that specific disease. Most, if not all, prognostic algorithms include some sort of classification of the primary tumour.[4-9] Therefore, the type of primary tumour - and the associated prognosis - is a key factor for selecting treatment, ranging from best supportive care to extensive spinal surgery.

The prognostication becomes even more challenging when the type of primary tumour is unknown, as in the case when metastatic spine disease is the initial manifestation of malignancy (IMM). When the patient presents with symptoms of epidural spinal cord compression, there is a limited time for further investigations before a decision of treatment. As the duration of neurological impairment is one of the factors predicting the neurological outcome, surgery (if applicable) should take place as soon as possible under the safest possible circumstances.[10]

The frequency of metastatic spine disease as IMM in surgically treated cohorts varies between different studies. In the widely-cited randomized controlled trial (RCT) by Patchell et al, 5 out of 50 surgically treated patients (10%) had an unknown primary tumour (UPT). The AO Spine Study published 2016 included 142 surgically treated patients and 17 of those (12 %) had UPT. In a more recent study by Wang et al with 448 surgically treated patients during a

For peer review only - http://bmjopen.bmj.com/site/about/guidelines.xhtml

period of 17 years, 15% patients presented with UPT. Other studies report up to 20% of UPT among patients treated surgically. [1 2 11]

Previous studies show varying results regarding survival after surgery for spinal metastatic disease for patients with UPT compared to known primary tumour (KPT). A study published by Quraishi et al in 2014, retrospectively evaluating a cohort of 285 patients of which 17 had UPT, reported no significant difference in survival between the two groups but a "trend towards shorter survival" in the UPT group.[12] A more recent study from Park et al showed that patients with spinal metastasis as IMM had longer survival than patients with known primary tumour, but the cases span from a period of 20 years.[13]. In the study by Wang et al from 2012, the UPT group had a longer median survival than the cohort as a whole (11.4 months vs 6.8 months).[14]

Considering these somewhat conflicting results, we decided to perform a survival analysis of a cohort of patients treated surgically due to spinal metastatic disease at Uppsala University Hospital 2006-2016. A part of this cohort has been investigated earlier by this group in a study analyzing the precision of predictive scores.[15]

Materials & methods

Study design

This is a longitudinal retrospective cohort study of consecutively treated patients with spinal metastatic disease. It is approved by the Regional Ethical Review Board in Uppsala (reference 2012/133).

For peer review only - http://bmjopen.bmj.com/site/about/guidelines.xhtml

For peer review only

Does knowledge of the primary tumour affect survival…

Setting

The study was performed in Sweden, where virtually all cancer patients are treated in the public healthcare. Uppsala University Hospital is one of seven university hospitals in Sweden and referral centre for about 2,1 million people.

Swespine, a national register for spine surgery, is maintained to monitor and develop the quality of spine surgery clinics. Swespine was established in 1993 and has a coverage of 49 of 50 clinics performing spine surgery in Sweden. A module for spinal metastatic disease was added to Swespine in 2006.

Participants

Patients 18 years or older undergoing surgery due to spinal metastatic disease at Uppsala University Hospital between January 1st 2006 and December 31st 2016 and registered in Swespine.

Individual patient history, including data on primary tumour, was obtained from the medical records at Uppsala University Hospital. The records are linked to the Swedish Population Registry, containing data on date of death if applicable.

Patient and Public Involvement

Patients or the public were not involved in the design, or conduct, or reporting, or dissemination plans of our research.

For peer review only - http://bmjopen.bmj.com/site/about/guidelines.xhtml

Does knowledge of the primary tumour affect survival…

Variables

Survival

Survival after surgery is defined as the time difference from the date of surgery, as reported to Swespine, and the date of death as recorded in the Swedish Population Register. All patients defined as alive in this study were alive as of January 25th 2021.

Type of tumour

Data on primary tumour is entered by the surgeon in Swespine. Available choices are known/unknown primary tumour and if known is chosen, a subset of eight choices becomes available. Data on tumour type in cases with unknown primary tumour is derived from the histopathology report, filed in the medical records, on the tissue sample collected during surgery. As a quality control, cases registered in Swespine as UPT were verified with the medical records to eliminate “false positive” UPT cases.

Indications and types of surgery

In Swespine, the surgeon reports the indication for surgery as well as the type of surgery. Possible indications according to the register are neurologic deficit, progressive deformity, pain or any combination thereof. Type of surgery is registered in terms of the presence of anterior/posterior decompression and the use of anterior/posterior implants.

For peer review only - http://bmjopen.bmj.com/site/about/guidelines.xhtml

Data Sources / measurement

A dataset was provided from Swespine including all patients undergoing surgery due to spinal metastatic disease in Sweden 2006-2016. Patients coded as treated at Uppsala University Hospital were selected for inclusion in the study. The data was cross-referenced with the medical records and the Swedish population register, using the personal identification number (PIN) for each record. (Figure 1).

Bias

There is most probably a selection bias as patients who are selected to undergo surgery generally have a better performance status and/or prognosis than patients selected to undergo non-surgical treatment. On the other hand, the risk for selection bias regarding registration in Swespine and thus inclusion in this study is negligible as registration in quality registers is regulated by law and is not at the discretion of the patient or the clinician.

Study size

The study size was determined by the number of patients with spinal metastatic disease being treated at Uppsala University Hospital and registered in Swespine 2006-2016. No power calculations were made prior to the study.

Statistical methods

IBM SPSS Statistics version 26 was used for all statistical calculations. The survival analysis was made with the Kaplan Meier Method and the Breslow test was used to test the statistical significance in the difference in survival between the two groups. The patient characteristics preoperatively were compared using Chi^2-test.

For peer review only - http://bmjopen.bmj.com/site/about/guidelines.xhtml

Linkage

Each entry in Swespine is labelled with the personal identification number (PIN) of the patient. The PIN was used to access the medical records including data on survival from the database at Uppsala University Hospital. Data from the medical records was manually entered into an anonymized database of the cases, stored in a secure location.

Results

393 patients registered in Swespine met the inclusion criteria and were selected for further analysis. 270 were males, 123 females and the mean age at the time of surgery was 67.3 years (range 23-91 years). 271 of the patients had a known primary tumour (KPT) verified in the medical records while 122 had unknown primary tumour at the time of surgery. There was a statistically significant lower percentage of males in the UPT group but no difference regarding age between the two groups. The level of missing data on smoking status was too high to draw any conclusions. (Table 1)

Data point	**Known primary tumour (KPT) (n=271)**	**Unknown primary tumour (UPT) (n=122)**	P-value
Mean age, years	67.5	66.9	0.47
Male sex	196 (72%)	74 (61%)	0.02
Known smokers	*11/139*	*9/61*	-

Table 1. Patient characteristics

For peer review only - http://bmjopen.bmj.com/site/about/guidelines.xhtml

For peer review only

Survival

The median estimated survival time after surgery for patients in the KPT group was 7.4 months (CI 6.0-8.7) and mean estimated survival was 21.6 months (CI 17.2-26.0). For patients in the UPT group, the median estimated survival was 15.6 months (CI 7.5-23.7) and mean estimated survival was 48.1 months (CI 37.3-59.0). After comparing the two groups with a Breslow test (Generalized Wilcoxon), the p-value was 0.001 and the difference in survival was considered as statistically significant. (Figure 2)

Tumour types

The most common primary tumour in the KPT group was prostate cancer, followed by lung cancer and blood, which in most cases means myeloma/plasmacytoma or lymphoma. In the UPT group, blood was the most common specified origin followed by lung cancer. The group "others" is a heterogenous group where unidentified tumours account for the majority. (Tables 2-3)

**KPT (as known before surgery)**	**n**	**%**
Prostate	100	37%
Blood	34	13%
Lung	28	10%
Renal	26	10%
Breast	19	7%
Gastrointestinal	16	6%
Thyroid	4	2%
Others	44	16%

For peer review only - http://bmjopen.bmj.com/site/about/guidelines.xhtml

Total	271	

Table 2. Distribution of primary tumours as known before surgery.

**UPT, result after histopathology**	**n**	**%**
Prostate	7	6%
Blood	43	35%
Lung	15	12%
Renal	1	1%
Breast	9	7%
Gastrointestinal	10	8%
Thyroid	1	1%
Others	36	30%
Total	122	

Table 3. Distribution of primary tumours types in the UPT group, according to histopathology reports after surgery.

Indications for surgery

Indications and types of surgery for the KPT and UPT groups were equally distributed. Neurologic deficit was the most common indication for surgery while posterior decompression with posterior implants (posterior stabilization) was the most common surgical procedure in both groups. (Tables 4,5)

**Indication for surgery**	**KPT group *n***	**KPT group %**	**UPT group *n***	**UPT group %**

For peer review only

Neurologic deficit (A)	161	59%	71	58%
Pain (B)	42	16%	12	10%
Progressive deformity (C)	2	1%	3	3%
A+B	27	10%	17	14%
A+C	0	0%	2	2%
B+C	2	1%	1	1%
A+B+C	6	2%	0	0%
Missing data	31	11%	16	1%
Total	271		70	

Table 4. Indications for surgery, as reported to Swespine.

For peer review only

**Type of surgery**	**KPT group *n***	**KPT group %**	**UPT group *n***	**UPT group %**	**Missing data *n***
Posterior decompression	219	81%	97	80%	6
Posterior implant	213	79%	94	77%	8
Anterior decompression	35	13%	19	16%	30

Anterior implant	42	15%	22	18%	17

Table 5. Types of surgery, as reported to Swespine.

Discussion

This is, to the best of our knowledge, one of the largest single-center cohorts with surgically treated patients in recent time. The single-center cohort study design facilitates access to medical records and reliable survival data, thanks to the linkage to the Swedish Population Register.

The distribution of primary tumours in this study is different from other studies. The prospective AO Spine Study by Fehlings et al published in 2016 excluded patients with known hematologic malignancies, but had three other tumour groups (lung, kidney, breast) ahead of prostate, which is the most common KPT in our Swedish material.[2] One possible explanation might be geographical, as the study by Wang et al performed in neighbouring Denmark has prostate cancer as the most common KPT followed by breast and kidney.[14]

There are several possible explanations to the longer survival in the UPT group compared to the KPT group. Firstly, the distribution of malignancies is different. In the UPT group, there is a higher rate of myeloma and lymphoma, cancer types known to respond very well to radiation. If the primary tumour had been known at the time of surgery, acute radiation rather than decompressive surgery would probably have been an option for some of those patients. The lack availability of emergency radiation treatment during weekends at referring hospitals

For peer review only - http://bmjopen.bmj.com/site/about/guidelines.xhtml

is another possible reason for the high amount of surgically treated cases in the group, underlining the need for access to acute radiation, preferably within 24 hours.[16]

Another explanation for the longer survival in the UPT group is that the tumour is treatment naïve and the field of oncologic treatment is still open. Furthermore, patients with spinal metastasis as IMM have per definition a state of health not causing an earlier urgent investigation of a possible disseminated cancer which suggests a good general health status.

One possible weakness of this study is the lack of control group of non-surgically treated patients, especially since there is a possible selection bias among patients deemed fit to undergo spine surgery. Reliable reporting of other outcomes, such as complications and the impact of health-related quality of life after surgery, is another possible field of improvement.

Nevertheless, the findings of our study suggest that patients with spinal metastatic disease as IMM should not be withheld from surgical treatment based on the fact that the primary tumour is unknown. The decision on whether to perform extensive spine surgery or not should be based on other factors, underlining the need for reliable tools for decision making. We suggest that further research in the field of surgical treatment of spinal metastatic should focus on other outcomes than survival, such as quality of life and complications after treatment.

Generalisability

The results of this study do not apply to all patients with spinal metastatic disease, due to the heterogeneity between different types of cancer.

For peer review only - http://bmjopen.bmj.com/site/about/guidelines.xhtml

Footnotes

Author Contributions

CC designed the study, drafted the manuscript and made the statistical analyses. CO was involved in the study design and reviewed the manuscript. YR was involved in the study design, wrote the ethical board application and reviewed the manuscript.

Funding

This research received no specific grant from any funding agency in the public, commercial or not-for-profit sectors.

Competing interests

Dr. Carrwik and Dr. Robinson have nothing to disclose. Dr. Olerud reports personal fees from Johnson & Johnson (paid speaker on course) outside the submitted work.

Ethics approval

This study was approved by the Regional Ethical Review Board in Uppsala (reference 2012/133).

Data availability statement

Source data provided by Swespine and from medical records stored by Region Uppsala. Swespine is available to researchers subject to application (http://www.swespine.se).

Figure legends

Figure 1. Flow-chart for inclusion and exclusion in the study.

Figure 2. Kaplan Meier chart comparing survival in the UPT and KPT groups.

For peer review only - http://bmjopen.bmj.com/site/about/guidelines.xhtml

Does knowledge of the primary tumour affect survival…

References

1. Patchell RA, Tibbs PA, Regine WF, et al. Direct decompressive surgical resection in the treatment of spinal cord compression caused by metastatic cancer: a randomised trial. *The Lancet* 2005;366(9486):643-48. doi: 10.1016/s0140-6736(05)66954-1
2. Fehlings MG, Nater A, Tetreault L, et al. Survival and Clinical Outcomes in Surgically Treated Patients With Metastatic Epidural Spinal Cord Compression: Results of the Prospective Multicenter AOSpine Study. *J Clin Oncol* 2016;34(3):268-76. doi: 10.1200/JCO.2015.61.9338
3. Dea N, Versteeg AL, Sahgal A, et al. Metastatic Spine Disease: Should Patients With Short Life Expectancy Be Denied Surgical Care? An International Retrospective Cohort Study. *Neurosurgery* 2020;87(2):303-11. doi: 10.1093/neuros/nyz472 [published Online First: 2019/11/07]
4. Tokuhashi Y, Matsuzaki H, Toriyama S, et al. Scoring system for the preoperative evaluation of metastatic spine tumor prognosis. *Spine (Phila Pa 1976)* 1990;15(11):1110-3. doi: 10.1097/00007632-199011010-00005 [published Online First: 1990/11/01]
5. Tokuhashi Y, Matsuzaki H, Oda H, et al. A revised scoring system for preoperative evaluation of metastatic spine tumor prognosis. *Spine (Phila Pa 1976)* 2005;30(19):2186-91. doi: 10.1097/01.brs.0000180401.06919.a5 [published Online First: 2005/10/06]
6. Bauer HC, Wedin R. Survival after surgery for spinal and extremity metastases. Prognostication in 241 patients. *Acta orthopaedica Scandinavica* 1995;66(2):143-6. doi: 10.3109/17453679508995508 [published Online First: 1995/04/01]
7. Tomita K, Kawahara N, Kobayashi T, et al. Surgical strategy for spinal metastases. *Spine (Phila Pa 1976)* 2001;26(3):298-306. doi: 10.1097/00007632-200102010-00016 [published Online First: 2001/02/27]
8. Ghori AK, Leonard DA, Schoenfeld AJ, et al. Modeling 1-year survival after surgery on the metastatic spine. *Spine J* 2015;15(11):2345-50. doi: 10.1016/j.spinee.2015.06.061 [published Online First: 2015/07/15]
9. Laufer I, Rubin DG, Lis E, et al. The NOMS framework: approach to the treatment of spinal metastatic tumors. *Oncologist* 2013;18(6):744-51. doi: 10.1634/theoncologist.2012-0293
10. Laufer I, Zuckerman SL, Bird JE, et al. Predicting Neurologic Recovery after Surgery in Patients with Deficits Secondary to MESCC: Systematic Review. *Spine (Phila Pa 1976)* 2016;41 Suppl 20(Suppl 20):S224-s30. doi: 10.1097/brs.0000000000001827 [published Online First: 2016/10/19]
11. Schiff D, O'Neill BP, Suman VJ. Spinal epidural metastasis as the initial manifestation of malignancy: clinical features and diagnostic approach. *Neurology* 1997;49(2):452-6. doi: 10.1212/wnl.49.2.452 [published Online First: 1997/08/01]
12. Quraishi NA, Ramoutar D, Sureshkumar D, et al. Metastatic spinal cord compression as a result of the unknown primary tumour. *European Spine Journal* 2014;23(7):1502-07. doi: 10.1007/s00586-014-3274-2
13. Park JS, Park SJ, Lee CS. Incidence and prognosis of patients with spinal metastasis as the initial manifestation of malignancy: analysis of 338 patients undergoing surgical treatment. *Bone Joint J* 2019;101-b(11):1379-84. doi: 10.1302/0301-620x.101b11.Bjj-2018-1600.R2 [published Online First: 2019/11/02]

For peer review only - http://bmjopen.bmj.com/site/about/guidelines.xhtml

14. Wang M, Bunger CE, Li H, et al. Predictive value of Tokuhashi scoring systems in spinal metastases, focusing on various primary tumor groups: evaluation of 448 patients in the Aarhus spinal metastases database. *Spine (Phila Pa 1976)* 2012;37(7):573-82. doi: 10.1097/BRS.0b013e31822bd6b0 [published Online First: 2011/07/29]
15. Carrwik C, Olerud C, Robinson Y. Predictive Scores Underestimate Survival of Patients with Metastatic Spine Disease: A Retrospective Study of 315 Patients in Sweden. *Spine (Phila Pa 1976)* 2019 doi: 10.1097/brs.0000000000003289 [published Online First: 2019/10/28]
16. Yeo R, Campbell T, Fairchild A. Is Weekend Radiation Therapy Always Justified? *J Med Imaging Radiat Sci* 2012;43(1):38-42. doi: 10.1016/j.jmir.2011.08.001 [published Online First: 2012/03/01]

For peer review only

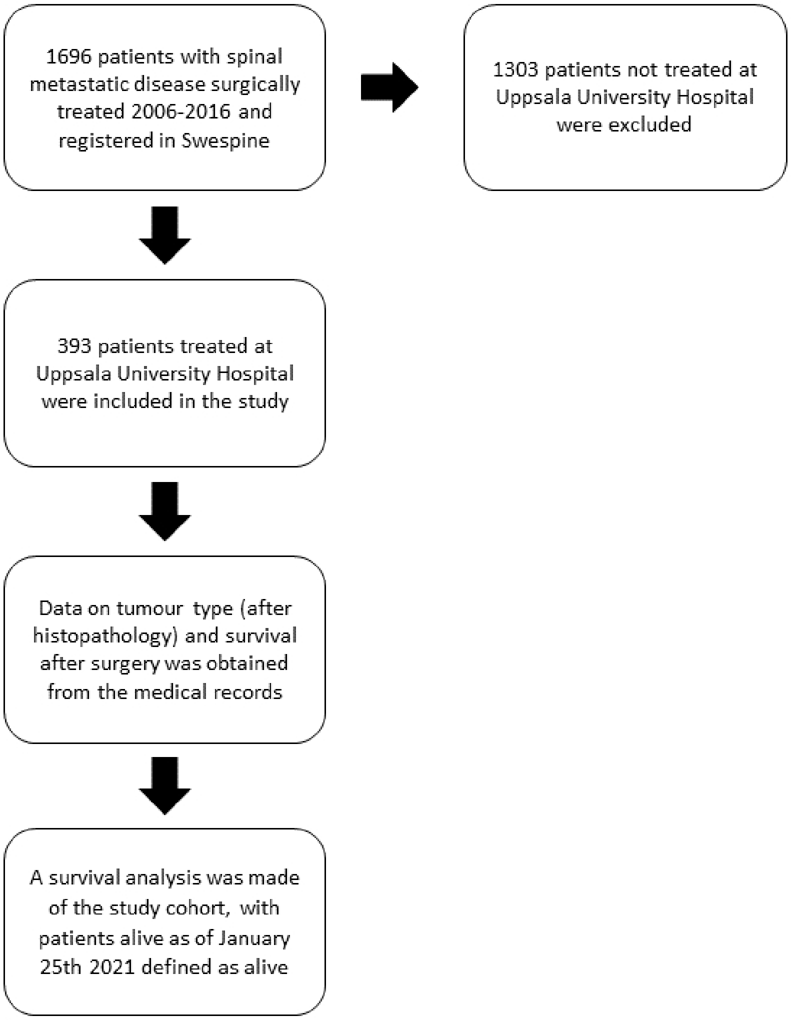

Flow-chart for inclusion and exclusion in the study.

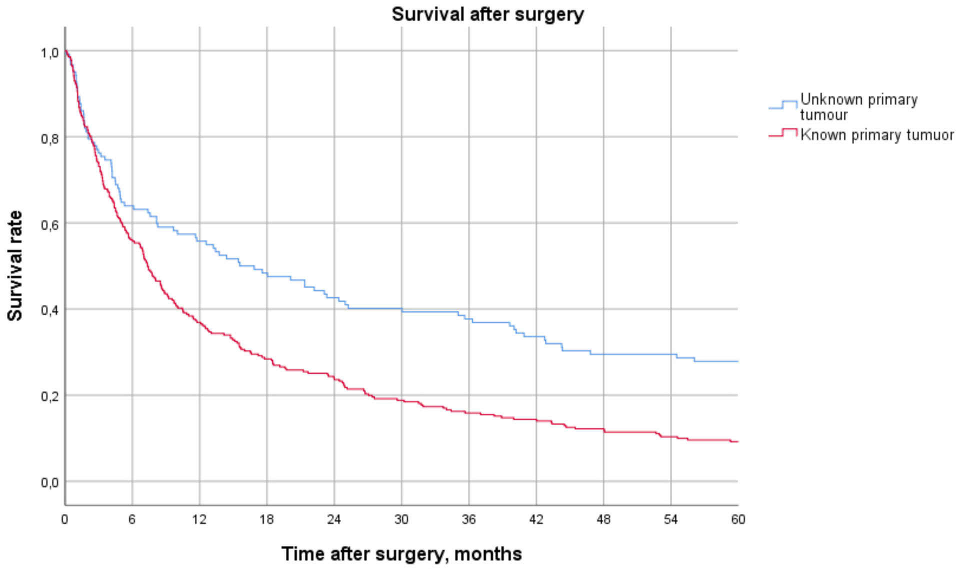

Kaplan Meier chart comparing survival in the UPT and KPT groups.

Reporting checklist for cohort study.

Based on the STROBE cohort guidelines.

Instructions to authors

Complete this checklist by entering the page numbers from your manuscript where readers will find each of the items listed below.

Your article may not currently address all the items on the checklist. Please modify your text to include the missing information. If you are certain that an item does not apply, please write "n/a" and provide a short explanation.

Upload your completed checklist as an extra file when you submit to a journal.

In your methods section, say that you used the STROBE cohortreporting guidelines, and cite them as:

von Elm E, Altman DG, Egger M, Pocock SJ, Gotzsche PC, Vandenbroucke JP. The Strengthening the Reporting of Observational Studies in Epidemiology (STROBE) Statement: guidelines for reporting observational studies.

For peer review only

		Reporting Item	Page Number
**Title and abstract**			
Title	#1a	Indicate the study's design with a commonly used term in the title or the abstract	1
Abstract	#1b	Provide in the abstract an informative and balanced summary	2

		of what was done and what was found	
**Introduction**			
Background / rationale	#2	Explain the scientific background and rationale for the investigation being reported	4
Objectives	#3	State specific objectives, including any prespecified hypotheses	5
**Methods**			
Study design	#4	Present key elements of study design early in the paper	5
Setting	#5	Describe the setting, locations, and relevant dates, including periods of recruitment, exposure, follow-up, and data collection	6
Eligibility criteria	#6a	Give the eligibility criteria, and the sources and methods of selection of participants. Describe methods of follow-up.	6
Eligibility criteria	#6b	For matched studies, give matching criteria and number of exposed and unexposed	6
Variables	#7	Clearly define all outcomes, exposures, predictors, potential confounders, and effect modifiers. Give diagnostic criteria, if applicable	7
Data sources / measurement	#8	For each variable of interest give sources of data and details of methods of assessment (measurement). Describe comparability of assessment methods if there is more than one group. Give information separately for for exposed and unexposed groups if applicable.	8

For peer review only

Bias	#9	Describe any efforts to address potential sources of bias	8
Study size	#10	Explain how the study size was arrived at	8
Quantitative variables	#11	Explain how quantitative variables were handled in the analyses. If applicable, describe which groupings were chosen, and why	7
Statistical methods	#12a	Describe all statistical methods, including those used to control for confounding	
8			
Statistical methods	#12b	Describe any methods used to examine subgroups and interactions	8
Statistical methods	#12c	Explain how missing data were addressed	12
Statistical methods	#12d	If applicable, explain how loss to follow-up was addressed	12
Statistical methods	#12e	Describe any sensitivity analyses	
12			
**Results**			
Participants	#13a	Report numbers of individuals at each stage of study—eg numbers potentially eligible, examined for eligibility, confirmed eligible, included in the study, completing follow-up, and analysed. Give information separately for for exposed and	9

For peer review only

		unexposed groups if applicable.	
Participants	#13b	Give reasons for non-participation at each stage	12
Participants	#13c	Consider use of a flow diagram	
8			
Descriptive data	#14a	Give characteristics of study participants (eg demographic, clinical, social) and information on exposures and potential confounders. Give information separately for exposed and unexposed groups if applicable.	6
Descriptive data	#14b	Indicate number of participants with missing data for each variable of interest	
11			
Descriptive data	#14c	Summarise follow-up time (eg, average and total amount)	
8			
Outcome data	#15	Report numbers of outcome events or summary measures over time. Give information separately for exposed and unexposed groups if applicable.	
7			
Main results	#16a	Give unadjusted estimates and, if applicable, confounder-adjusted estimates and their precision (eg, 95% confidence interval). Make clear which confounders were adjusted for and why they were included	9
Main results	#16b	Report category boundaries when continuous variables were	9

For peer review only

		categorized	
Main results	#16c	If relevant, consider translating estimates of relative risk into absolute risk for a meaningful time period	
9			
Other analyses	#17	Report other analyses done—eg analyses of subgroups and interactions, and sensitivity analyses	9
**Discussion**			
Key results	#18	Summarise key results with reference to study objectives	10
Limitations	#19	Discuss limitations of the study, taking into account sources of potential bias or imprecision. Discuss both direction and magnitude of any potential bias.	13
Interpretation	#20	Give a cautious overall interpretation considering objectives, limitations, multiplicity of analyses, results from similar studies, and other relevant evidence.	14
Generalisability	#21	Discuss the generalisability (external validity) of the study results	14
**Other Information**			
Funding	#22	Give the source of funding and the role of the funders for the present study and, if applicable, for the original study on which the present article is based	15

For peer review only

The STROBE checklist is distributed under the terms of the Creative Commons Attribution License CC-BY. This checklist was completed on 22. February 2021 using https://www.goodreports.org/, a tool made by the EQUATOR Network in collaboration with Penelope.ai

For peer review only

BMJ Open

Does knowledge of the primary tumour affect survival after surgery for spinal metastatic disease? A retrospective longitudinal cohort study

Journal:	*BMJ Open*
Manuscript ID	bmjopen-2021-050538.R1
Article Type:	Original research
Date Submitted by the Author:	24-Jun-2021
Complete List of Authors:	Carrwik, Christian; Uppsala University, Department of Surgical Sciences<br>Olerud, Claes; Uppsala University, Department of Surgical Sciences<br>Robinson, Yohan; Uppsala University, Department of Surgical Sciences; Armed Forces Centre for Defence Medicine, Department of Research and Development
**Primary Subject Heading**:	Surgery
Secondary Subject Heading:	Surgery
Keywords:	Spine < ORTHOPAEDIC & TRAUMA SURGERY, Neurosurgery < SURGERY, Orthopaedic oncology < ORTHOPAEDIC & TRAUMA SURGERY

SCHOLARONE™
Manuscripts

I, the Submitting Author has the right to grant and does grant on behalf of all authors of the Work (as defined in the below author licence), an exclusive licence and/or a non-exclusive licence for contributions from authors who are: i) UK Crown employees; ii) where BMJ has agreed a CC-BY licence shall apply, and/or iii) in accordance with the terms applicable for US Federal Government officers or employees acting as part of their official duties; on a worldwide, perpetual, irrevocable, royalty-free basis to BMJ Publishing Group Ltd ("BMJ") its licensees and where the relevant Journal is co-owned by BMJ to the co-owners of the Journal, to publish the Work in this journal and any other BMJ products and to exploit all rights, as set out in our licence.

The Submitting Author accepts and understands that any supply made under these terms is made by BMJ to the Submitting Author unless you are acting as an employee on behalf of your employer or a postgraduate student of an affiliated institution which is paying any applicable article publishing charge ("APC") for Open Access articles. Where the Submitting Author wishes to make the Work available on an Open Access basis (and intends to pay the relevant APC), the terms of reuse of such Open Access shall be governed by a Creative Commons licence – details of these licences and which Creative Commons licence will apply to this Work are set out in our licence referred to above.

Other than as permitted in any relevant BMJ Author's Self Archiving Policies, I confirm this Work has not been accepted for publication elsewhere, is not being considered for publication elsewhere and does not duplicate material already published. I confirm all authors consent to publication of this Work and authorise the granting of this licence.

For peer review only

Title

Does knowledge of the primary tumour affect survival after surgery for spinal metastatic disease? A retrospective longitudinal cohort study

Authors

Christian Carrwik MD (corresponding author), Claes Olerud MD PhD, Yohan Robinson MD PhD MBA

Christian Carrwik MD (corresponding author)
christian.carrwik@surgsci.uu.se
Department of Surgical Sciences
Uppsala University
SE-751 85 Uppsala
Sweden
Phone +46-(0)18-611 00 00

Claes Olerud MD PhD
claes.olerud@surgsci.uu.se
Department of Surgical Sciences
Uppsala University

Yohan Robinson MD PhD MBA

For peer review only

For peer review only - http://bmjopen.bmj.com/site/about/guidelines.xhtml

Does knowledge of the primary tumour affect survival…

yohan.robinson@gu.se
Department of Surgical Sciences
Uppsala University
Sweden
Department of Research and Development
Armed Forces Centre for Defence Medicine
Gothenburg
Sweden
Institute of Clinical Sciences
Sahlgrenska Academy, Gothenburg University
Gothenburg
Sweden

For peer review only

Abstract

Objectives: To compare survival after surgery for patients with spinal metastatic disease with known primary tumour (KPT) versus patients with unknown primary tumour (UPT).

Participants: 393 patients 18 years or older (270 men, 123 women, mean age 67.3 years) undergoing surgery at Uppsala University Hospital in Sweden due to spinal metastatic disease 2006-2016. 271 patients (69%) had a KPT at the time of surgery and 122 (31%) had an UPT.

Interventions: Decompressive and/or stabilizing spine surgery due to spinal metastatic disease.

Primary outcome: Survival (median and mean) after surgery.

Results: The estimated median survival time after surgery for patients with KPT was 7.4 months (CI 6.0-8.7) and mean survival time 21.6 months (CI 17.2-26.0). For patients with

Does knowledge of the primary tumour affect survival…

UPT, the median estimated survival time after surgery was 15.6 months (CI 7.5-23.7) and the mean survival time 48.1 months (CI 37.3-59.0) (Breslow, p=0.001). Unknown primary cancer was a positive predictor of survival after surgery (Cox regression, HR = 0.58, CI 0.46-0.73).

Conclusion: In this study, patients with spinal metastasis and UPT had a longer expected survival after surgery compared to patients with KPT. This suggests that patients with UPT and spinal metastasis should not be withheld from surgery only based on the fact that the primary tumour is unknown.

Key words: Tumour, spine, metastases, cancer, surgery, survival

Word count: 2308 words.

Strengths and limitations of this study

- Large single-center cohort with at least four years follow-up time
- Reliable survival data with linkage to the Swedish Population Register
- Includes results of biopsies of the unknown primary tumours
- No control group with patients treated only non-surgically
- Lacks reliable data on quality of life and neurologic outcome after surgery

Introduction

The evolution of oncological treatment and longer expected survival has lowered the threshold for surgical intervention among patients with metastatic spine disease in recent

For peer review only - http://bmjopen.bmj.com/site/about/guidelines.xhtml

decades. In several studies, decompressive surgery (often in combination with surgical stabilization) has been found to be beneficial for patients with an expected survival of at least three months.[1 2] In a recent study by Dea et al, this time frame has been challenged and the authors suggest that the extent of the surgery and patient specific factors rather than expected survival should be guiding in the decision-making process.[3]

The prognosis for the individual patient depends on several factors, including the patient's performance status and the extent of generalization of the malignancy. One of the most important factors is the type of primary tumour and the underlying prognosis for that specific disease. Most, if not all, prognostic algorithms include some sort of classification of the primary tumour.[4-9] Therefore, the type of primary tumour - and the associated prognosis - is a key factor for selecting treatment, ranging from best supportive care to extensive spinal surgery.

The prognostication becomes even more challenging when the type of primary tumour is unknown, as in the case when metastatic spine disease is the initial manifestation of malignancy (IMM). When the patient presents with symptoms of epidural spinal cord compression, there is a limited time for further investigations before a decision of treatment. As the duration of neurological impairment is one of the factors predicting the neurological outcome, surgery (if applicable) should take place as soon as possible under the safest possible circumstances.[10]

The frequency of metastatic spine disease as IMM in surgically treated cohorts varies between different studies. In the widely-cited randomized controlled trial (RCT) by Patchell et al, 5 out of 50 surgically treated patients (10%) had an unknown primary tumour (UPT). The AO

For peer review only - http://bmjopen.bmj.com/site/about/guidelines.xhtml

Spine Study published 2016 included 142 surgically treated patients and 17 of those (12 %) had UPT. In a more recent study by Wang et al with 448 surgically treated patients during a period of 17 years, 15% patients presented with UPT. Other studies report up to 20% of UPT among patients treated surgically. [1 2 11]

Previous studies show varying results regarding survival after surgery for spinal metastatic disease for patients with UPT compared to known primary tumour (KPT). A study published by Quraishi et al in 2014, retrospectively evaluating a cohort of 285 patients of which 17 had UPT, reported no significant difference in survival between the two groups but a "trend towards shorter survival" in the UPT group.[12] A more recent study from Park et al showed that patients with spinal metastasis as IMM had longer survival than patients with known primary tumour, but the cases span from a period of 20 years.[13]. In the study by Wang et al from 2012, the UPT group had a longer median survival than the cohort as a whole (11.4 months vs 6.8 months).[14]

Considering these somewhat conflicting results, we decided to perform a survival analysis of a cohort of patients treated surgically due to spinal metastatic disease at Uppsala University Hospital 2006-2016. A part of this cohort has been investigated earlier by this group in a study analyzing the precision of predictive scores.[15]

Materials & methods

Study design

This is a longitudinal retrospective cohort study of consecutively treated patients with spinal

For peer review only - http://bmjopen.bmj.com/site/about/guidelines.xhtml

metastatic disease. It is approved by the Regional Ethical Review Board in Uppsala (reference 2012/133).

Setting

The study was performed in Sweden, where virtually all cancer patients are treated in the public healthcare. Uppsala University Hospital is one of seven university hospitals in Sweden and referral centre for about 2,1 million people.

Swespine, a national register for spine surgery, is maintained to monitor and develop the quality of spine surgery clinics. Swespine was established in 1993 and has a coverage of 49 of 50 clinics performing spine surgery in Sweden. A module for spinal metastatic disease was added to Swespine in 2006.

Participants

Patients 18 years or older undergoing surgery due to spinal metastatic disease at Uppsala University Hospital between January 1st 2006 and December 31st 2016 and registered in Swespine.

Individual patient history, including data on primary tumour, was obtained from the medical records at Uppsala University Hospital. The records are linked to the Swedish Population Registry, containing data on date of death if applicable.

For peer review only - http://bmjopen.bmj.com/site/about/guidelines.xhtml

Does knowledge of the primary tumour affect survival…

Patient and Public Involvement

Patients or the public were not involved in the design, or conduct, or reporting, or dissemination plans of our research.

Variables

Survival

Survival after surgery is defined as the time difference from the date of surgery, as reported to Swespine, and the date of death as recorded in the Swedish Population Register. All patients defined as alive in this study were alive as of January 25th 2021.

Type of tumour

Data on primary tumour is entered by the surgeon in Swespine. Available choices are known/unknown primary tumour and if known is chosen, a subset of eight choices becomes available. Data on tumour type in cases with unknown primary tumour is derived from the histopathology report, filed in the medical records, on the tissue sample collected during surgery. As a quality control, cases registered in Swespine as UPT were verified with the medical records to eliminate "false positive" UPT cases.

Indications and types of surgery

In Swespine, the surgeon reports the indication for surgery as well as the type of surgery. Possible indications according to the register are neurologic deficit, progressive deformity,

For peer review only - http://bmjopen.bmj.com/site/about/guidelines.xhtml

pain or any combination thereof. Type of surgery is registered in terms of the presence of anterior/posterior decompression and the use of anterior/posterior implants.

Data Sources / measurement

A dataset was provided from Swespine including all patients undergoing surgery due to spinal metastatic disease in Sweden 2006-2016. Patients coded as treated at Uppsala University Hospital were selected for inclusion in the study. The data was cross-referenced with the medical records and the Swedish population register, using the personal identification number (PIN) for each record. (Figure 1)

Bias

There is most probably a selection bias as patients who are selected to undergo surgery generally have a better performance status and/or prognosis than patients selected to undergo non-surgical treatment. On the other hand, the risk for selection bias regarding registration in Swespine and thus inclusion in this study is negligible as registration in quality registers is regulated by law and is not at the discretion of the patient or the clinician.

Study size

The study size was determined by the number of patients with spinal metastatic disease being treated at Uppsala University Hospital and registered in Swespine 2006-2016. No power calculations were made prior to the study.

For peer review only

Statistical methods

IBM SPSS Statistics version 26 was used for all statistical calculations. The survival analysis was made with the Kaplan Meier Method and the Breslow test was used to test the statistical significance in the difference in survival between the two groups. The patient characteristics preoperatively were compared using a chi^2-test.

Using the Cox proportional hazards regression method, the contribution of multiple covariates to survival was analyzed and presented as hazard ratio (HR) with 95% CI and probability p. Besides an unknown primary tumour, patient age and confirmed primary tumour type in biopsy were identified as relevant covariates for survival. Since we assumed that oncological advancement had improved patient survival in general in recent decades, decade of admission was included as a covariate in the model. Covariates were treated as categorical variables.

Linkage

Each entry in Swespine is labelled with the personal identification number (PIN) of the patient. The PIN was used to access the medical records including data on survival from the database at Uppsala University Hospital. Data from the medical records was manually entered into an anonymized database of the cases, stored in a secure location.

Results

393 patients registered in Swespine met the inclusion criteria and were selected for further analysis. 270 were males, 123 females and the mean age at the time of surgery was 67.3 years (range 23-91 years). 271 of the patients had a known primary tumour (KPT) verified in the

For peer review only - http://bmjopen.bmj.com/site/about/guidelines.xhtml

medical records while 122 had unknown primary tumour at the time of surgery. There was a statistically significant lower percentage of males in the UPT group but no difference regarding age between the two groups. The level of missing data on smoking status was too high to draw any conclusions. (Table 1)

Data point	**Known primary tumour (KPT) (n=271)**	**Unknown primary tumour (UPT) (n=122)**	P-value
Mean age, years	67.5	66.9	0.47
Male sex	196 (72%)	74 (61%)	0.02
Active smoker (yes/no)	*11/139*	*9/61*	-

Table 1. Patient characteristics

Survival

The median estimated survival time after surgery for patients in the KPT group was 7.4 months (CI 6.0-8.7) and mean estimated survival was 21.6 months (CI 17.2-26.0). For patients in the UPT group, the median estimated survival was 15.6 months (CI 7.5-23.7) and mean estimated survival was 48.1 months (CI 37.3-59.0). After comparing the two groups with a Breslow test (Generalized Wilcoxon), the p-value was 0.001 and the difference in survival was considered as statistically significant. Regarding true survival data, 58 of the patients (15%) survived at least five years after surgery and ten years after surgery 15 patients (4%) were still alive. As of January 25th 2021, 39 of the patients (10%) in the cohort were alive. (Figure 2)

For peer review only - http://bmjopen.bmj.com/site/about/guidelines.xhtml

Tumour types

The most common primary tumour in the KPT group was prostate cancer, followed by lung cancer and blood, which in most cases means myeloma/plasmacytoma or lymphoma. In the UPT group, blood was the most common specified origin followed by lung cancer. The group “others” is a heterogenous group where still unidentified tumours, even after histopathology, account for the majority. The difference in distribution of primary tumours between the KPT and UPT groups reached statistical significance ($p<0.05$) in four categories. (Table 2)

**Tumour type per group**	**KPT (as known before surgery) n**	**KPT (as known before surgery) %**	**UPT (after histopathology) n**	**UPT (after histopathology) %**	**p-value**
Prostate	100	37%	7	6%	<0.0001
Blood	34	13%	43	35%	<0.0001
Lung	28	10%	15	12%	0.56
Renal	26	10%	1	1%	0.001
Breast	19	7%	9	7%	0.90
Gastrointestinal	16	6%	10	8%	0.40
Thyroid	4	2%	1	1%	0.59
Others	44	16%	36	30%	0.03

For peer review only - http://bmjopen.bmj.com/site/about/guidelines.xhtml

Does knowledge of the primary tumour affect survival…

Total	271		122		

Table 2. Distribution of primary tumours in the KPT and UPT groups.

Tumors originating from the blood had the lowest hazard-ratio of the investigated tumour types, while lung tumours had the highest hazard-ratio. (Table 3).

Covariate	Categories	N Deaths/Subjects	N Person-months	Univariate HR (95% CI)	Univariate p	Multivariate HR (95% C.I.)	Multivariate p
Primary tumour							
	known	257/271	5276	1.00 (Ref)		1.00 (Ref)	
	unknown	97/122	4784	0.58 (0.46-0.73)	<0.001	0.69 (0.53-0.90)	0.005
Age							
	<50 years	15/22	1440	1.00 (Ref)	0.24	1.00 (Ref)	<0.001
	50-64 years	104/127	4132	HR 1.97 (1.14-3.41)	0.15	2.82 (1.62-4.90)	<0.001
	65-80 years	192/201	4016	HR 1.93 (1.13-3.30)	0.16	3.28 (1.90-5.66)	<0.001
	>80 years	43/43	473	HR 2.55 (1.40-4.64)	0.002	5.55 (2.99-10.30)	<0.001
Primary tumour in biopsy							
	Prostate	106/107	1791	1.00 (Ref)		1.00 (Ref)	
	Blood	49/77	449	0.30 (0.21-0.42)	<0.001	0.42 (0.28-0.62)	<0.001
	Lung	43/43	504	1.64 (1.15-2.35)	0.007	2.53 (1.72-3.71)	<0.001
	Renal	25/27	453	0.95 (0.61-1.46)	0.80	1.02 (0.65-1.59)	0.94
	Breast	28/28	806	0.75 (0.49-1.14)	0.18	0.96 (0.62-1.47)	0.84
	Gastrointestinal	22/26	522	0.86 (0.54-1.36)	0.52	1.50 (0.91-2.48)	0.11
	Thyroid	5/5	204	0.59 (0.24-1.45)	0.30	0.70 (0.28-1,72)	0.44
	Others	76/80	1663	1.17 (0.87-1.57)	0.30	1.61 (1.18-2.21)	0.03
Decade of surgery							
	2000s	185/196	4643	1.00 (Ref)		1.00 (Ref)	
	2010s	169/197	5417	0.94 (0.76-1.16)	0.54	0.89 (0.71-1.11)	0.30

Table 3. Univariate and multivariate Cox regression analysis of covariates impacting survival after surgery.

For peer review only - http://bmjopen.bmj.com/site/about/guidelines.xhtml

Indications for surgery

Neurologic deficit was the most common indication for surgery while posterior decompression with posterior implants (posterior stabilization) was the most common surgical procedure in both groups. The distributions of indications for surgery and types of surgery were not statistically significant between the two groups. (Tables 4,5)

**Indication for surgery**	**KPT group *n***	**KPT group %**	**UPT group *n***	**UPT group %**	**p-value**
Neurologic deficit (A)	161	59%	71	58%	0.82
Pain (B)	42	16%	12	10%	0.13
Progressive deformity (C)	2	1%	3	3%	0.16
A+B	27	10%	17	14%	0.25
A+C	0	0%	2	2%	(-)
B+C	2	1%	1	1%	0.93
A+B+C	6	2%	0	0%	(-)
Missing data	31	11%	16	1%	0.64
Total	271		122		

Table 4. Indications for surgery, as reported to Swespine. In the groups marked with (-) there were too few observations to calculate a p-value.

**Type of surgery**	**KPT group *n***	**KPT group %**	**UPT group *n***	**UPT group %**	**Missing data *n***	**p-value**

For peer review only

Does knowledge of the primary tumour affect survival…

Posterior decompression	219	81%	97	80%	6	0.76
Posterior implant	213	79%	94	77%	8	0.73
Anterior decompression	35	13%	19	16%	30	0.49
Anterior implant	42	15%	22	18%	17	0.53

Table 5. Types of surgery, as reported to Swespine.

Discussion

This is, to the best of our knowledge, one of the largest single-center cohorts with surgically treated patients in recent time. The single-center cohort study design facilitates access to medical records and reliable survival data, thanks to the linkage to the Swedish Population Register.

The distribution of primary tumours in this study is different from other studies. The prospective AO Spine Study by Fehlings et al published in 2016 excluded patients with known hematologic malignancies, but had three other tumour groups (lung, kidney, breast) ahead of prostate, which is the most common KPT in our Swedish material.[2] One possible

For peer review only - http://bmjopen.bmj.com/site/about/guidelines.xhtml

explanation might be geographical, as the study by Wang et al performed in neighbouring Denmark has prostate cancer as the most common KPT followed by breast and kidney.[14]

There are several possible explanations to the longer survival in the UPT group compared to the KPT group. Firstly, the distribution of malignancies is different. In the UPT group, there is a higher rate of myeloma and lymphoma, cancer types known to respond very well to radiation. If the primary tumour had been known at the time of surgery, acute radiation rather than decompressive surgery would probably have been an option for some of those patients. The lack of availability of emergency radiation treatment during weekends at referring hospitals is another possible reason for the high amount of surgically treated cases in the group, underlining the need for access to acute radiation, preferably within 24 hours.[16]

Another explanation for the longer survival in the UPT group is that the tumour is treatment naïve and the field of oncologic treatment is still open. Furthermore, patients with spinal metastasis as IMM have per definition a state of health not causing an earlier urgent investigation of a possible disseminated cancer which suggests a good general health status.

One possible weakness of this study is the lack of control group of non-surgically treated patients, especially since there is a possible selection bias among patients deemed fit to undergo spine surgery. Reliable reporting of other outcomes, such as complications and the impact of health-related quality of life after surgery, is another possible field of improvement.

Nevertheless, the findings of our study suggest that patients with spinal metastatic disease as IMM should not be withheld from surgical treatment based on the fact that the primary tumour is unknown. The decision on whether to perform extensive spine surgery or not should

For peer review only - http://bmjopen.bmj.com/site/about/guidelines.xhtml

be based on other factors, underlining the need for reliable tools for decision making. We suggest that further research in the field of surgical treatment of spinal metastatic should focus on other outcomes than survival, such as quality of life and complications after treatment.

Generalisability

The results of this study do not apply to all patients with spinal metastatic disease, due to the heterogeneity between different types of cancer.

Footnotes

Author Contributions

CC designed the study, drafted the manuscript and made the statistical analyses. CO was involved in the study design and reviewed the manuscript. YR was involved in the study design, wrote the ethical board application and reviewed the manuscript.

Funding

This research received no specific grant from any funding agency in the public, commercial or not-for-profit sectors.

Competing interests

Dr. Carrwik and Dr. Robinson have nothing to disclose. Dr. Olerud reports personal fees from Johnson & Johnson (paid speaker on course) outside the submitted work.

For peer review only - http://bmjopen.bmj.com/site/about/guidelines.xhtml

Does knowledge of the primary tumour affect survival…

Ethics approval

This study was approved by the Regional Ethical Review Board in Uppsala (reference 2012/133).

Data availability statement

Source data provided by Swespine and from medical records stored by Region Uppsala.

Swespine is available to researchers subject to application (http://www.swespine.se).

Figure legends

Figure 1. Flow-chart for inclusion and exclusion in the study.

Figure 2. Kaplan Meier chart comparing survival in the UPT and KPT groups.

References

1. Patchell RA, Tibbs PA, Regine WF, et al. Direct decompressive surgical resection in the treatment of spinal cord compression caused by metastatic cancer: a randomised trial. *The Lancet* 2005;366(9486):643-48. doi: 10.1016/s0140-6736(05)66954-1
2. Fehlings MG, Nater A, Tetreault L, et al. Survival and Clinical Outcomes in Surgically Treated Patients With Metastatic Epidural Spinal Cord Compression: Results of the Prospective Multicenter AOSpine Study. *J Clin Oncol* 2016;34(3):268-76. doi: 10.1200/JCO.2015.61.9338
3. Dea N, Versteeg AL, Sahgal A, et al. Metastatic Spine Disease: Should Patients With Short Life Expectancy Be Denied Surgical Care? An International Retrospective Cohort Study. *Neurosurgery* 2020;87(2):303-11. doi: 10.1093/neuros/nyz472 [published Online First: 2019/11/07]
4. Tokuhashi Y, Matsuzaki H, Toriyama S, et al. Scoring system for the preoperative evaluation of metastatic spine tumor prognosis. *Spine (Phila Pa 1976)* 1990;15(11):1110-3. doi: 10.1097/00007632-199011010-00005 [published Online First: 1990/11/01]
5. Tokuhashi Y, Matsuzaki H, Oda H, et al. A revised scoring system for preoperative evaluation of metastatic spine tumor prognosis. *Spine (Phila Pa 1976)* 2005;30(19):2186-91. doi: 10.1097/01.brs.0000180401.06919.a5 [published Online First: 2005/10/06]

For peer review only - http://bmjopen.bmj.com/site/about/guidelines.xhtml

6. Bauer HC, Wedin R. Survival after surgery for spinal and extremity metastases. Prognostication in 241 patients. *Acta orthopaedica Scandinavica* 1995;66(2):143-6. doi: 10.3109/17453679508995508 [published Online First: 1995/04/01]
7. Tomita K, Kawahara N, Kobayashi T, et al. Surgical strategy for spinal metastases. *Spine (Phila Pa 1976)* 2001;26(3):298-306. doi: 10.1097/00007632-200102010-00016 [published Online First: 2001/02/27]
8. Ghori AK, Leonard DA, Schoenfeld AJ, et al. Modeling 1-year survival after surgery on the metastatic spine. *Spine J* 2015;15(11):2345-50. doi: 10.1016/j.spinee.2015.06.061 [published Online First: 2015/07/15]
9. Laufer I, Rubin DG, Lis E, et al. The NOMS framework: approach to the treatment of spinal metastatic tumors. *Oncologist* 2013;18(6):744-51. doi: 10.1634/theoncologist.2012-0293
10. Laufer I, Zuckerman SL, Bird JE, et al. Predicting Neurologic Recovery after Surgery in Patients with Deficits Secondary to MESCC: Systematic Review. *Spine (Phila Pa 1976)* 2016;41 Suppl 20(Suppl 20):S224-s30. doi: 10.1097/brs.0000000000001827 [published Online First: 2016/10/19]
11. Schiff D, O'Neill BP, Suman VJ. Spinal epidural metastasis as the initial manifestation of malignancy: clinical features and diagnostic approach. *Neurology* 1997;49(2):452-6. doi: 10.1212/wnl.49.2.452 [published Online First: 1997/08/01]
12. Quraishi NA, Ramoutar D, Sureshkumar D, et al. Metastatic spinal cord compression as a result of the unknown primary tumour. *European Spine Journal* 2014;23(7):1502-07. doi: 10.1007/s00586-014-3274-2
13. Park JS, Park SJ, Lee CS. Incidence and prognosis of patients with spinal metastasis as the initial manifestation of malignancy: analysis of 338 patients undergoing surgical treatment. *Bone Joint J* 2019;101-b(11):1379-84. doi: 10.1302/0301-620x.101b11.Bjj-2018-1600.R2 [published Online First: 2019/11/02]
14. Wang M, Bunger CE, Li H, et al. Predictive value of Tokuhashi scoring systems in spinal metastases, focusing on various primary tumor groups: evaluation of 448 patients in the Aarhus spinal metastases database. *Spine (Phila Pa 1976)* 2012;37(7):573-82. doi: 10.1097/BRS.0b013e31822bd6b0 [published Online First: 2011/07/29]
15. Carrwik C, Olerud C, Robinson Y. Predictive Scores Underestimate Survival of Patients with Metastatic Spine Disease: A Retrospective Study of 315 Patients in Sweden. *Spine (Phila Pa 1976)* 2019 doi: 10.1097/brs.0000000000003289 [published Online First: 2019/10/28]
16. Yeo R, Campbell T, Fairchild A. Is Weekend Radiation Therapy Always Justified? *J Med Imaging Radiat Sci* 2012;43(1):38-42. doi: 10.1016/j.jmir.2011.08.001 [published Online First: 2012/03/01]

For peer review only - http://bmjopen.bmj.com/site/about/guidelines.xhtml

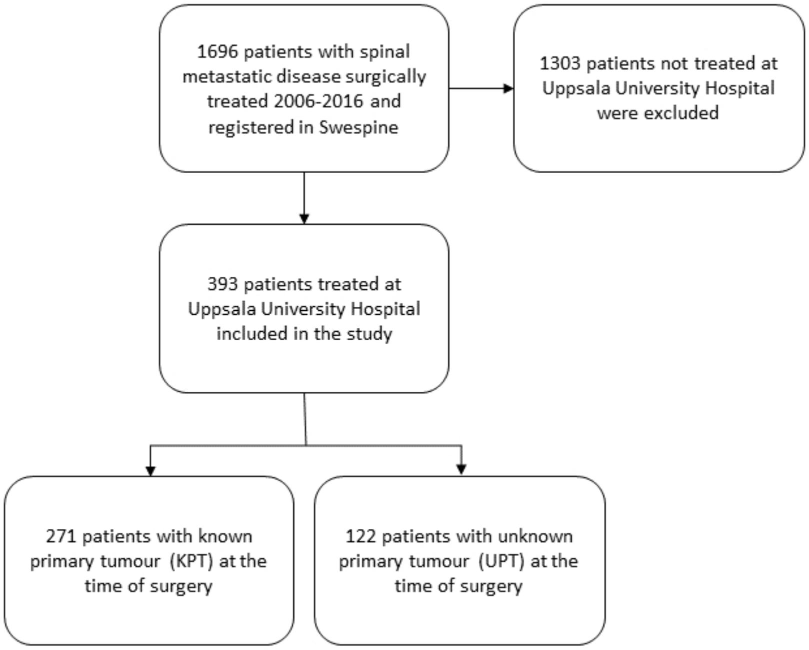

Flow-chart for inclusion and exclusion in the study.

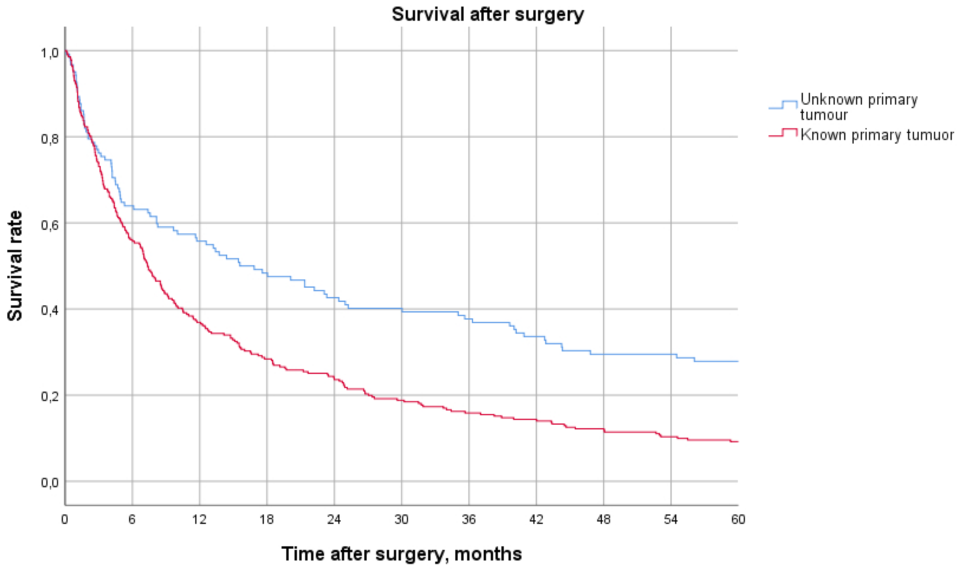

Kaplan Meier chart comparing survival in the UPT and KPT groups.

Reporting checklist for cohort study.

Based on the STROBE cohort guidelines.

Instructions to authors

Complete this checklist by entering the page numbers from your manuscript where readers will find each of the items listed below.

Your article may not currently address all the items on the checklist. Please modify your text to include the missing information. If you are certain that an item does not apply, please write "n/a" and provide a short explanation.

Upload your completed checklist as an extra file when you submit to a journal.

In your methods section, say that you used the STROBE cohortreporting guidelines, and cite them as:

von Elm E, Altman DG, Egger M, Pocock SJ, Gotzsche PC, Vandenbroucke JP. The Strengthening the Reporting of Observational Studies in Epidemiology (STROBE) Statement: guidelines for reporting observational studies.

		Reporting Item	Page Number
**Title and abstract**			
Title	#1a	Indicate the study’s design with a commonly used term in the title or the abstract	1
Abstract	#1b	Provide in the abstract an informative and balanced summary	2

For peer review only

		of what was done and what was found	
**Introduction**			
Background / rationale	#2	Explain the scientific background and rationale for the investigation being reported	4
Objectives	#3	State specific objectives, including any prespecified hypotheses	5
**Methods**			
Study design	#4	Present key elements of study design early in the paper	5
Setting	#5	Describe the setting, locations, and relevant dates, including periods of recruitment, exposure, follow-up, and data collection	6
Eligibility criteria	#6a	Give the eligibility criteria, and the sources and methods of selection of participants. Describe methods of follow-up.	6
Eligibility criteria	#6b	For matched studies, give matching criteria and number of exposed and unexposed	6
Variables	#7	Clearly define all outcomes, exposures, predictors, potential confounders, and effect modifiers. Give diagnostic criteria, if applicable	7
Data sources / measurement	#8	For each variable of interest give sources of data and details of methods of assessment (measurement). Describe comparability of assessment methods if there is more than one group. Give information separately for for exposed and unexposed groups if applicable.	8

For peer review only

Bias	#9	Describe any efforts to address potential sources of bias	8
Study size	#10	Explain how the study size was arrived at	8
Quantitative variables	#11	Explain how quantitative variables were handled in the analyses. If applicable, describe which groupings were chosen, and why	7
Statistical methods	#12a	Describe all statistical methods, including those used to control for confounding	
8			
Statistical methods	#12b	Describe any methods used to examine subgroups and interactions	8
Statistical methods	#12c	Explain how missing data were addressed	12
Statistical methods	#12d	If applicable, explain how loss to follow-up was addressed	12
Statistical methods	#12e	Describe any sensitivity analyses	
12			

Results

Participants	#13a	Report numbers of individuals at each stage of study—eg numbers potentially eligible, examined for eligibility, confirmed eligible, included in the study, completing follow-up, and analysed. Give information separately for for exposed and	9

For peer review only

		unexposed groups if applicable.	
Participants	#13b	Give reasons for non-participation at each stage	12
Participants	#13c	Consider use of a flow diagram	
8			
Descriptive data	#14a	Give characteristics of study participants (eg demographic, clinical, social) and information on exposures and potential confounders. Give information separately for exposed and unexposed groups if applicable.	6
Descriptive data	#14b	Indicate number of participants with missing data for each variable of interest	
11			
Descriptive data	#14c	Summarise follow-up time (eg, average and total amount)	
8			
Outcome data	#15	Report numbers of outcome events or summary measures over time. Give information separately for exposed and unexposed groups if applicable.	
7			
Main results	#16a	Give unadjusted estimates and, if applicable, confounder-adjusted estimates and their precision (eg, 95% confidence interval). Make clear which confounders were adjusted for and why they were included	9
Main results	#16b	Report category boundaries when continuous variables were	9

For peer review only

		categorized	
Main results	#16c	If relevant, consider translating estimates of relative risk into absolute risk for a meaningful time period	
9			
Other analyses	#17	Report other analyses done—eg analyses of subgroups and interactions, and sensitivity analyses	9
**Discussion**			
Key results	#18	Summarise key results with reference to study objectives	10
Limitations	#19	Discuss limitations of the study, taking into account sources of potential bias or imprecision. Discuss both direction and magnitude of any potential bias.	13
Interpretation	#20	Give a cautious overall interpretation considering objectives, limitations, multiplicity of analyses, results from similar studies, and other relevant evidence.	14
Generalisability	#21	Discuss the generalisability (external validity) of the study results	14
**Other Information**			
Funding	#22	Give the source of funding and the role of the funders for the present study and, if applicable, for the original study on which the present article is based	15

For peer review only

The STROBE checklist is distributed under the terms of the Creative Commons Attribution License CC-BY. This checklist was completed on 22. February 2021 using https://www.goodreports.org/, a tool made by the EQUATOR Network in collaboration with Penelope.ai

For peer review only

BMJ Open

Does knowledge of the primary tumour affect survival after surgery for spinal metastatic disease? A retrospective longitudinal cohort study

Journal:	*BMJ Open*
Manuscript ID	bmjopen-2021-050538.R2
Article Type:	Original research
Date Submitted by the Author:	07-Aug-2021
Complete List of Authors:	Carrwik, Christian; Uppsala University, Department of Surgical Sciences<br>Olerud, Claes; Uppsala University, Department of Surgical Sciences<br>Robinson, Yohan; Uppsala University, Department of Surgical Sciences; Armed Forces Centre for Defence Medicine, Department of Research and Development
**Primary Subject Heading**:	Surgery
Secondary Subject Heading:	Surgery
Keywords:	Spine < ORTHOPAEDIC & TRAUMA SURGERY, Neurosurgery < SURGERY, Orthopaedic oncology < ORTHOPAEDIC & TRAUMA SURGERY

SCHOLARONE™
Manuscripts

I, the Submitting Author has the right to grant and does grant on behalf of all authors of the Work (as defined in the below author licence), an exclusive licence and/or a non-exclusive licence for contributions from authors who are: i) UK Crown employees; ii) where BMJ has agreed a CC-BY licence shall apply, and/or iii) in accordance with the terms applicable for US Federal Government officers or employees acting as part of their official duties; on a worldwide, perpetual, irrevocable, royalty-free basis to BMJ Publishing Group Ltd ("BMJ") its licensees and where the relevant Journal is co-owned by BMJ to the co-owners of the Journal, to publish the Work in this journal and any other BMJ products and to exploit all rights, as set out in our licence.

The Submitting Author accepts and understands that any supply made under these terms is made by BMJ to the Submitting Author unless you are acting as an employee on behalf of your employer or a postgraduate student of an affiliated institution which is paying any applicable article publishing charge ("APC") for Open Access articles. Where the Submitting Author wishes to make the Work available on an Open Access basis (and intends to pay the relevant APC), the terms of reuse of such Open Access shall be governed by a Creative Commons licence – details of these licences and which Creative Commons licence will apply to this Work are set out in our licence referred to above.

Other than as permitted in any relevant BMJ Author's Self Archiving Policies, I confirm this Work has not been accepted for publication elsewhere, is not being considered for publication elsewhere and does not duplicate material already published. I confirm all authors consent to publication of this Work and authorise the granting of this licence.

For peer review only

Title

Does knowledge of the primary tumour affect survival after surgery for spinal metastatic disease? A retrospective longitudinal cohort study

Authors

Christian Carrwik MD (corresponding author), Claes Olerud MD PhD, Yohan Robinson MD PhD MBA

Christian Carrwik MD (corresponding author)
christian.carrwik@surgsci.uu.se
Department of Surgical Sciences
Uppsala University
SE-751 85 Uppsala
Sweden
Phone +46-(0)18-611 00 00

Claes Olerud MD PhD
claes.olerud@surgsci.uu.se
Department of Surgical Sciences
Uppsala University

Yohan Robinson MD PhD MBA

For peer review only

For peer review only - http://bmjopen.bmj.com/site/about/guidelines.xhtml

yohan.robinson@gu.se
Department of Surgical Sciences
Uppsala University
Sweden
Department of Research and Development
Armed Forces Centre for Defence Medicine
Gothenburg
Sweden
Institute of Clinical Sciences
Sahlgrenska Academy, Gothenburg University
Gothenburg
Sweden

Abstract

Objectives: To compare survival after surgery for patients with spinal metastatic disease with known primary tumour (KPT) versus patients with unknown primary tumour (UPT).

Participants: 393 patients 18 years or older (270 men, 123 women, mean age 67.3 years) undergoing surgery at Uppsala University Hospital in Sweden due to spinal metastatic disease 2006-2016. 271 patients (69%) had a KPT at the time of surgery and 122 (31%) had an UPT.

Interventions: Decompressive and/or stabilizing spine surgery due to spinal metastatic disease.

Primary outcome: Survival (median and mean) after surgery.

Results: The estimated median survival time after surgery for patients with KPT was 7.4 months (CI 6.0-8.7) and mean survival time 21.6 months (CI 17.2-26.0). For patients with

For peer review only - http://bmjopen.bmj.com/site/about/guidelines.xhtml

For peer review only

UPT, the median estimated survival time after surgery was 15.6 months (CI 7.5-23.7) and the mean survival time 48.1 months (CI 37.3-59.0) (Breslow, p=0.001). Unknown primary cancer was a positive predictor of survival after surgery (Cox regression, HR = 0.58, CI 0.46-0.73).

Conclusion: In this study, patients with spinal metastasis and UPT had a longer expected survival after surgery compared to patients with KPT. This suggests that patients with UPT and spinal metastasis should not be withheld from surgery only based on the fact that the primary tumour is unknown.

Key words: Tumour, spine, metastases, cancer, surgery, survival

Word count: 2292 words.

Strengths and limitations of this study

- Large single-center cohort with at least four years follow-up time
- Reliable survival data with linkage to the Swedish Population Register
- Includes results of biopsies of the unknown primary tumours
- No control group with patients treated only non-surgically
- Lacks reliable data on quality of life and neurologic outcome after surgery

Introduction

The evolution of oncological treatment and longer expected survival has lowered the threshold for surgical intervention among patients with metastatic spine disease in recent

For peer review only

decades. In several studies, decompressive surgery (often in combination with surgical stabilization) has been found to be beneficial for patients with an expected survival of at least three months.[1 2] In a recent study by Dea et al, this time frame has been challenged and the authors suggest that the extent of the surgery and patient specific factors rather than expected survival should be guiding in the decision-making process.[3]

The prognosis for the individual patient depends on several factors, including the patient's performance status and the extent of generalization of the malignancy. One of the most important factors is the type of primary tumour and the underlying prognosis for that specific disease. Most, if not all, prognostic algorithms include some sort of classification of the primary tumour.[4-9] Therefore, the type of primary tumour - and the associated prognosis - is a key factor for selecting treatment, ranging from best supportive care to extensive spinal surgery.

The prognostication becomes even more challenging when the type of primary tumour is unknown, as in the case when metastatic spine disease is the initial manifestation of malignancy (IMM). When the patient presents with symptoms of epidural spinal cord compression, there is a limited time for further investigations before a decision of treatment. As the duration of neurological impairment is one of the factors predicting the neurological outcome, surgery (if applicable) should take place as soon as possible under the safest possible circumstances.[10]

The frequency of metastatic spine disease as IMM in surgically treated cohorts varies between different studies. In the widely-cited randomized controlled trial (RCT) by Patchell et al, 5 out of 50 surgically treated patients (10%) had an unknown primary tumour (UPT). The AO

For peer review only - http://bmjopen.bmj.com/site/about/guidelines.xhtml

Spine Study published 2016 included 142 surgically treated patients and 17 of those (12 %) had UPT. In a more recent study by Wang et al with 448 surgically treated patients during a period of 17 years, 15% patients presented with UPT. Other studies report up to 20% of UPT among patients treated surgically. [1 2 11]

Previous studies show varying results regarding survival after surgery for spinal metastatic disease for patients with UPT compared to known primary tumour (KPT). A study published by Quraishi et al in 2014, retrospectively evaluating a cohort of 285 patients of which 17 had UPT, reported no significant difference in survival between the two groups but a “trend towards shorter survival” in the UPT group.[12] A more recent study from Park et al showed that patients with spinal metastasis as IMM had longer survival than patients with known primary tumour, but the cases span from a period of 20 years.[13]. In the study by Wang et al from 2012, the UPT group had a longer median survival than the cohort as a whole (11.4 months vs 6.8 months).[14]

Considering these somewhat conflicting results, we decided to perform a survival analysis of a cohort of patients treated surgically due to spinal metastatic disease at Uppsala University Hospital 2006-2016. A part of this cohort has been investigated earlier by this group in a study analyzing the precision of predictive scores.[15]

Materials & methods

Study design

This is a longitudinal retrospective cohort study of consecutively treated patients with spinal

For peer review only - http://bmjopen.bmj.com/site/about/guidelines.xhtml

metastatic disease. It is approved by the Regional Ethical Review Board in Uppsala (reference 2012/133).

Setting

The study was performed in Sweden, where virtually all cancer patients are treated in the public healthcare. Uppsala University Hospital is one of seven university hospitals in Sweden and referral centre for about 2,1 million people.

Swespine, a national register for spine surgery, is maintained to monitor and develop the quality of spine surgery clinics. Swespine was established in 1993 and has a coverage of 49 of 50 clinics performing spine surgery in Sweden. A module for spinal metastatic disease was added to Swespine in 2006.

Participants

Patients 18 years or older undergoing surgery due to spinal metastatic disease at Uppsala University Hospital between January 1st 2006 and December 31st 2016 and registered in Swespine.

Individual patient history, including data on primary tumour, was obtained from the medical records at Uppsala University Hospital. The records are linked to the Swedish Population Registry, containing data on date of death if applicable.

For peer review only - http://bmjopen.bmj.com/site/about/guidelines.xhtml

Patient and Public Involvement

Patients or the public were not involved in the design, or conduct, or reporting, or dissemination plans of our research.

Variables

Survival

Survival after surgery is defined as the time difference from the date of surgery, as reported to Swespine, and the date of death as recorded in the Swedish Population Register. All patients defined as alive in this study were alive as of January 25th 2021.

Type of tumour

Data on primary tumour is entered by the surgeon in Swespine. Available choices are known/unknown primary tumour and if known is chosen, a subset of eight choices becomes available. Data on tumour type in cases with unknown primary tumour is derived from the histopathology report, filed in the medical records, on the tissue sample collected during surgery. As a quality control, cases registered in Swespine as UPT were verified with the medical records to eliminate "false positive" UPT cases.

Indications and types of surgery

In Swespine, the surgeon reports the indication for surgery as well as the type of surgery. Possible indications according to the register are neurologic deficit, progressive deformity,

For peer review only - http://bmjopen.bmj.com/site/about/guidelines.xhtml

pain or any combination thereof. Type of surgery is registered in terms of the presence of anterior/posterior decompression and the use of anterior/posterior implants.

Data Sources / measurement

A dataset was provided from Swespine including all patients undergoing surgery due to spinal metastatic disease in Sweden 2006-2016. Patients coded as treated at Uppsala University Hospital were selected for inclusion in the study. The data was cross-referenced with the medical records and the Swedish population register, using the personal identification number (PIN) for each record. (Figure 1)

Bias

There is most probably a selection bias as patients who are selected to undergo surgery generally have a better performance status and/or prognosis than patients selected to undergo non-surgical treatment. On the other hand, the risk for selection bias regarding registration in Swespine and thus inclusion in this study is negligible as registration in quality registers is regulated by law and is not at the discretion of the patient or the clinician.

Study size

The study size was determined by the number of patients with spinal metastatic disease being treated at Uppsala University Hospital and registered in Swespine 2006-2016. No power calculations were made prior to the study.

For peer review only - http://bmjopen.bmj.com/site/about/guidelines.xhtml

Statistical methods

IBM SPSS Statistics version 26 was used for all statistical calculations. The survival analysis was made with the Kaplan Meier Method and the Breslow test was used to test the statistical significance in the difference in survival between the two groups. The patient characteristics preoperatively were compared using a chi^2-test.

Using the Cox proportional hazards regression method, the contribution of multiple covariates to survival was analyzed and presented as hazard ratio (HR) with 95% CI and probability p. Besides an unknown primary tumour, patient age and confirmed primary tumour type in biopsy were identified as relevant covariates for survival. Since we assumed that oncological advancement had improved patient survival in general in recent decades, decade of admission was included as a covariate in the model. Covariates were treated as categorical variables.

Linkage

Each entry in Swespine is labelled with the personal identification number (PIN) of the patient. The PIN was used to access the medical records including data on survival from the database at Uppsala University Hospital. Data from the medical records was manually entered into an anonymized database of the cases, stored in a secure location.

Results

393 patients registered in Swespine met the inclusion criteria and were selected for further analysis. 270 were males, 123 females and the mean age at the time of surgery was 67.3 years (range 23-91 years). 271 of the patients had a known primary tumour (KPT) verified in the

For peer review only
For peer review only - http://bmjopen.bmj.com/site/about/guidelines.xhtml

medical records while 122 had unknown primary tumour at the time of surgery. There was a statistically significant lower percentage of males in the UPT group but no difference regarding age between the two groups. The level of missing data on smoking status was too high to draw any conclusions. (Table 1)

Data point	**Known primary tumour (KPT) (n=271)**	**Unknown primary tumour (UPT) (n=122)**	P-value
Mean age, years	67.5	66.9	0.47
Male sex	196 (72%)	74 (61%)	0.02
Active smoker (yes/no)	*11/139*	*9/61*	-

Table 1. Patient characteristics

Survival

The median estimated survival time after surgery for patients in the KPT group was 7.4 months (CI 6.0-8.7) and mean estimated survival was 21.6 months (CI 17.2-26.0). For patients in the UPT group, the median estimated survival was 15.6 months (CI 7.5-23.7) and mean estimated survival was 48.1 months (CI 37.3-59.0). The estimated five-year survival was 9% in the KPT group and 28% in the UPT group. Estimated ten-year survival was 5% in the KPT group and 18% in the UPT group. After comparing the two groups with a Breslow test (Generalized Wilcoxon), the p-value was 0.001 and the difference in survival was considered as statistically significant. As of January 25th 2021, 39 of the patients (10%) in the cohort were alive. (Figure 2)

For peer review only - http://bmjopen.bmj.com/site/about/guidelines.xhtml

Tumour types

The most common primary tumour in the KPT group was prostate cancer, followed by lung cancer and blood, which in most cases means myeloma/plasmacytoma or lymphoma. In the UPT group, blood was the most common specified origin followed by lung cancer. The group “others” is a heterogenous group where still unidentified tumours, even after the postoperative histopathology, account for the majority. Cases with only non-specific pathology report results such as “squamous cells” are included here. The difference in distribution of primary tumours between the KPT and UPT groups reached statistical significance ($p<0.05$) in four categories.

(Table 2)

Tumour type per group	KPT (as known before surgery) n	KPT (as known before surgery) %	UPT (after histopathology) n	UPT (after histopathology) %	p-value
Prostate	100	37%	7	6%	<0.0001
Blood	34	13%	43	35%	<0.0001
Lung	28	10%	15	12%	0.56
Renal	26	10%	1	1%	0.001
Breast	19	7%	9	7%	0.90
Gastrointestinal	16	6%	10	8%	0.40

For peer review only - http://bmjopen.bmj.com/site/about/guidelines.xhtml

Thyroid	4	2%	1	1%	0.59
Others	44	16%	36	30%	0.03
Total	271		122		

Table 2. Distribution of primary tumours in the KPT and UPT groups.

Tumors originating from the blood had the lowest hazard-ratio of the investigated tumour types, while lung tumours had the highest hazard-ratio. (Table 3).

Covariate	Categories	N Deaths/Subjects	N Person-months	Univariate HR (95% CI)	p	Multivariate HR (95% C.I.)	p
Primary tumour							
	known	257/271	5276	1.00 (Ref)		1.00 (Ref)	
	unknown	97/122	4784	0.58 (0.46-0.73)	<0.001	0.69 (0.53-0.90)	0.005
Age							
	<50 years	15/22	1440	1.00 (Ref)	0.24	1.00 (Ref)	<0.001
	50-64 years	104/127	4132	HR 1.97 (1.14-3.41)	0.15	2.82 (1.62-4.90)	<0.001
	65-80 years	192/201	4016	HR 1.93 (1.13-3.30)	0.16	3.28 (1.90-5.66)	<0.001
	>80 years	43/43	473	HR 2.55 (1.40-4.64)	0.002	5.55 (2.99-10.30)	<0.001
Primary tumour in biopsy							
	Prostate	106/107	1791	1.00 (Ref)		1.00 (Ref)	
	Blood	49/77	449	0.30 (0.21-0.42)	<0.001	0.42 (0.28-0.62)	<0.001
	Lung	43/43	504	1.64 (1.15-2.35)	0.007	2.53 (1.72-3.71)	<0.001
	Renal	25/27	453	0.95 (0.61-1.46)	0.80	1.02 (0.65-1.59)	0.94
	Breast	28/28	806	0.75 (0.49-1.14)	0.18	0.96 (0.62-1.47)	0.84
	Gastrointestinal	22/26	522	0.86 (0.54-1.36)	0.52	1.50 (0.91-2.48)	0.11
	Thyroid	5/5	204	0.59 (0.24-1.45)	0.30	0.70 (0.28-1,72)	0.44
	Others	76/80	1663	1.17 (0.87-1.57)	0.30	1.61 (1.18-2.21)	0.03
Decade of surgery							
	2000s	185/196	4643	1.00 (Ref)		1.00 (Ref)	
	2010s	169/197	5417	0.94 (0.76-1.16)	0.54	0.89 (0.71-1.11)	0.30

Table 3. Univariate and multivariate Cox regression analysis of covariates impacting survival after surgery.

For peer review only - http://bmjopen.bmj.com/site/about/guidelines.xhtml

Indications for surgery

Neurologic deficit was the most common indication for surgery while posterior decompression with posterior implants (posterior stabilization) was the most common surgical procedure in both groups. The distributions of indications for surgery and types of surgery were not statistically significant between the two groups. (Tables 4,5)

**Indication for surgery**	**KPT group *n***	**KPT group %**	**UPT group *n***	**UPT group %**	**p-value**
Neurologic deficit (A)	161	59%	71	58%	0.82
Pain (B)	42	16%	12	10%	0.13
Progressive deformity (C)	2	1%	3	3%	0.16
A+B	27	10%	17	14%	0.25
A+C	0	0%	2	2%	(-)
B+C	2	1%	1	1%	0.93
A+B+C	6	2%	0	0%	(-)
Missing data	31	11%	16	1%	0.64
Total	271		122		

Table 4. Indications for surgery, as reported to Swespine. In the groups marked with (-) there were too few observations to calculate a p-value.

For peer review only - http://bmjopen.bmj.com/site/about/guidelines.xhtml

Does knowledge of the primary tumour affect survival…

**Type of surgery**	**KPT group *n***	**KPT group %**	**UPT group *n***	**UPT group %**	**Missing data *n***	**p-value**
Posterior decompression	219	81%	97	80%	6	0.76
Posterior implant	213	79%	94	77%	8	0.73
Anterior decompression	35	13%	19	16%	30	0.49
Anterior implant	42	15%	22	18%	17	0.53

Table 5. Types of surgery, as reported to Swespine.

Discussion

This is, to the best of our knowledge, one of the largest single-center cohorts with surgically treated patients in recent time. The single-center cohort study design facilitates access to medical records and reliable survival data, thanks to the linkage to the Swedish Population Register.

The distribution of primary tumours in this study is different from other studies. The prospective AO Spine Study by Fehlings et al published in 2016 excluded patients with

For peer review only - http://bmjopen.bmj.com/site/about/guidelines.xhtml

Does knowledge of the primary tumour affect survival…

known hematologic malignancies, but had three other tumour groups (lung, kidney, breast) ahead of prostate, which is the most common KPT in our Swedish material.[2] One possible explanation might be geographical, as the study by Wang et al performed in neighbouring Denmark has prostate cancer as the most common KPT followed by breast and kidney.[14]

There are several possible explanations to the longer survival in the UPT group compared to the KPT group. Firstly, the distribution of malignancies is different. In the UPT group, there is a higher rate of myeloma and lymphoma, cancer types known to respond very well to radiation. If the primary tumour had been known at the time of surgery, acute radiation rather than decompressive surgery would probably have been an option for some of those patients. The lack of availability of emergency radiation treatment during weekends at referring hospitals is another possible reason for the high amount of surgically treated cases in the group, underlining the need for access to acute radiation, preferably within 24 hours.[16]

Another explanation for the longer survival in the UPT group is that the tumour is treatment naïve and the field of oncologic treatment is still open. Furthermore, patients with spinal metastasis as IMM have per definition a state of health not causing an earlier urgent investigation of a possible disseminated cancer which suggests a good general health status.

One possible weakness of this study is the lack of control group of non-surgically treated patients, especially since there is a possible selection bias among patients deemed fit to undergo spine surgery. Reliable reporting of other outcomes, such as complications and the impact of health-related quality of life after surgery, is another possible field of improvement.

For peer review only - http://bmjopen.bmj.com/site/about/guidelines.xhtml

Does knowledge of the primary tumour affect survival…

Nevertheless, the findings of our study suggest that patients with spinal metastatic disease as IMM should not be withheld from surgical treatment based on the fact that the primary tumour is unknown. The decision on whether to perform extensive spine surgery or not should be based on other factors, underlining the need for reliable tools for decision making. We suggest that further research in the field of surgical treatment of spinal metastatic should focus on other outcomes than survival, such as quality of life and complications after treatment.

Generalisability

The results of this study do not apply to all patients with spinal metastatic disease, due to the heterogeneity between different types of cancer.

Footnotes

Author Contributions

CC designed the study, drafted the manuscript and made the statistical analyses. CO was involved in the study design and reviewed the manuscript. YR was involved in the study design, wrote the ethical board application and reviewed the manuscript.

Funding

This research received no specific grant from any funding agency in the public, commercial or not-for-profit sectors.

For peer review only

For peer review only - http://bmjopen.bmj.com/site/about/guidelines.xhtml

Competing interests

Dr. Carrwik and Dr. Robinson have nothing to disclose. Dr. Olerud reports personal fees from Johnson & Johnson (paid speaker on course) outside the submitted work.

Ethics approval

This study was approved by the Regional Ethical Review Board in Uppsala (reference 2012/133).

Data availability statement

Source data provided by Swespine and from medical records stored by Region Uppsala.

Swespine is available to researchers subject to application (http://www.swespine.se).

Figure legends

Figure 1. Flow-chart for inclusion and exclusion in the study.

Figure 2. Kaplan Meier chart comparing survival in the UPT and KPT groups.

References

1. Patchell RA, Tibbs PA, Regine WF, et al. Direct decompressive surgical resection in the treatment of spinal cord compression caused by metastatic cancer: a randomised trial. *The Lancet* 2005;366(9486):643-48. doi: 10.1016/s0140-6736(05)66954-1
2. Fehlings MG, Nater A, Tetreault L, et al. Survival and Clinical Outcomes in Surgically Treated Patients With Metastatic Epidural Spinal Cord Compression: Results of the Prospective Multicenter AOSpine Study. *J Clin Oncol* 2016;34(3):268-76. doi: 10.1200/JCO.2015.61.9338
3. Dea N, Versteeg AL, Sahgal A, et al. Metastatic Spine Disease: Should Patients With Short Life Expectancy Be Denied Surgical Care? An International Retrospective Cohort Study. *Neurosurgery* 2020;87(2):303-11. doi: 10.1093/neuros/nyz472 [published Online First: 2019/11/07]

For peer review only - http://bmjopen.bmj.com/site/about/guidelines.xhtml

4. Tokuhashi Y, Matsuzaki H, Toriyama S, et al. Scoring system for the preoperative evaluation of metastatic spine tumor prognosis. *Spine (Phila Pa 1976)* 1990;15(11):1110-3. doi: 10.1097/00007632-199011010-00005 [published Online First: 1990/11/01]
5. Tokuhashi Y, Matsuzaki H, Oda H, et al. A revised scoring system for preoperative evaluation of metastatic spine tumor prognosis. *Spine (Phila Pa 1976)* 2005;30(19):2186-91. doi: 10.1097/01.brs.0000180401.06919.a5 [published Online First: 2005/10/06]
6. Bauer HC, Wedin R. Survival after surgery for spinal and extremity metastases. Prognostication in 241 patients. *Acta orthopaedica Scandinavica* 1995;66(2):143-6. doi: 10.3109/17453679508995508 [published Online First: 1995/04/01]
7. Tomita K, Kawahara N, Kobayashi T, et al. Surgical strategy for spinal metastases. *Spine (Phila Pa 1976)* 2001;26(3):298-306. doi: 10.1097/00007632-200102010-00016 [published Online First: 2001/02/27]
8. Ghori AK, Leonard DA, Schoenfeld AJ, et al. Modeling 1-year survival after surgery on the metastatic spine. *Spine J* 2015;15(11):2345-50. doi: 10.1016/j.spinee.2015.06.061 [published Online First: 2015/07/15]
9. Laufer I, Rubin DG, Lis E, et al. The NOMS framework: approach to the treatment of spinal metastatic tumors. *Oncologist* 2013;18(6):744-51. doi: 10.1634/theoncologist.2012-0293
10. Laufer I, Zuckerman SL, Bird JE, et al. Predicting Neurologic Recovery after Surgery in Patients with Deficits Secondary to MESCC: Systematic Review. *Spine (Phila Pa 1976)* 2016;41 Suppl 20(Suppl 20):S224-s30. doi: 10.1097/brs.0000000000001827 [published Online First: 2016/10/19]
11. Schiff D, O'Neill BP, Suman VJ. Spinal epidural metastasis as the initial manifestation of malignancy: clinical features and diagnostic approach. *Neurology* 1997;49(2):452-6. doi: 10.1212/wnl.49.2.452 [published Online First: 1997/08/01]
12. Quraishi NA, Ramoutar D, Sureshkumar D, et al. Metastatic spinal cord compression as a result of the unknown primary tumour. *European Spine Journal* 2014;23(7):1502-07. doi: 10.1007/s00586-014-3274-2
13. Park JS, Park SJ, Lee CS. Incidence and prognosis of patients with spinal metastasis as the initial manifestation of malignancy: analysis of 338 patients undergoing surgical treatment. *Bone Joint J* 2019;101-b(11):1379-84. doi: 10.1302/0301-620x.101b11.Bjj-2018-1600.R2 [published Online First: 2019/11/02]
14. Wang M, Bunger CE, Li H, et al. Predictive value of Tokuhashi scoring systems in spinal metastases, focusing on various primary tumor groups: evaluation of 448 patients in the Aarhus spinal metastases database. *Spine (Phila Pa 1976)* 2012;37(7):573-82. doi: 10.1097/BRS.0b013e31822bd6b0 [published Online First: 2011/07/29]
15. Carrwik C, Olerud C, Robinson Y. Predictive Scores Underestimate Survival of Patients with Metastatic Spine Disease: A Retrospective Study of 315 Patients in Sweden. *Spine (Phila Pa 1976)* 2019 doi: 10.1097/brs.0000000000003289 [published Online First: 2019/10/28]
16. Yeo R, Campbell T, Fairchild A. Is Weekend Radiation Therapy Always Justified? *J Med Imaging Radiat Sci* 2012;43(1):38-42. doi: 10.1016/j.jmir.2011.08.001 [published Online First: 2012/03/01]

For peer review only

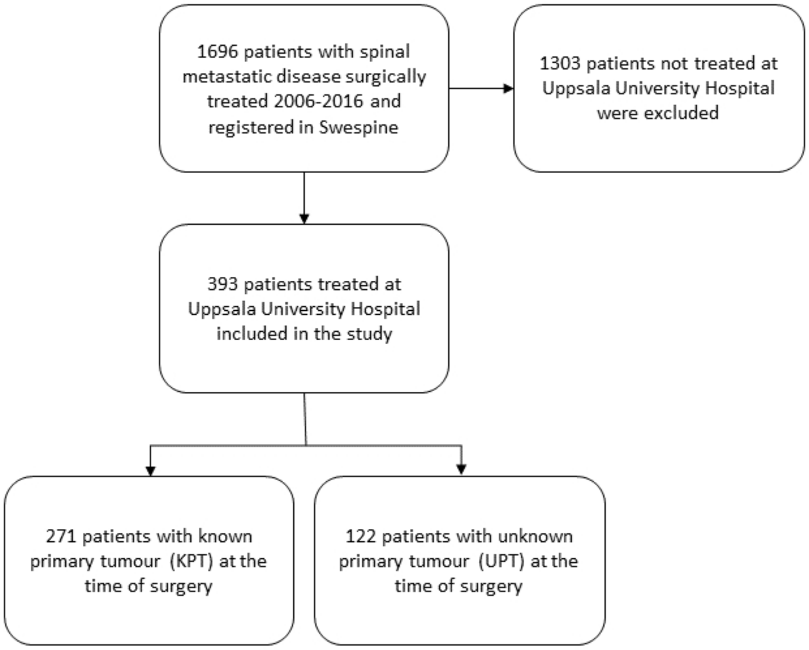

Flow-chart for inclusion and exclusion in the study.

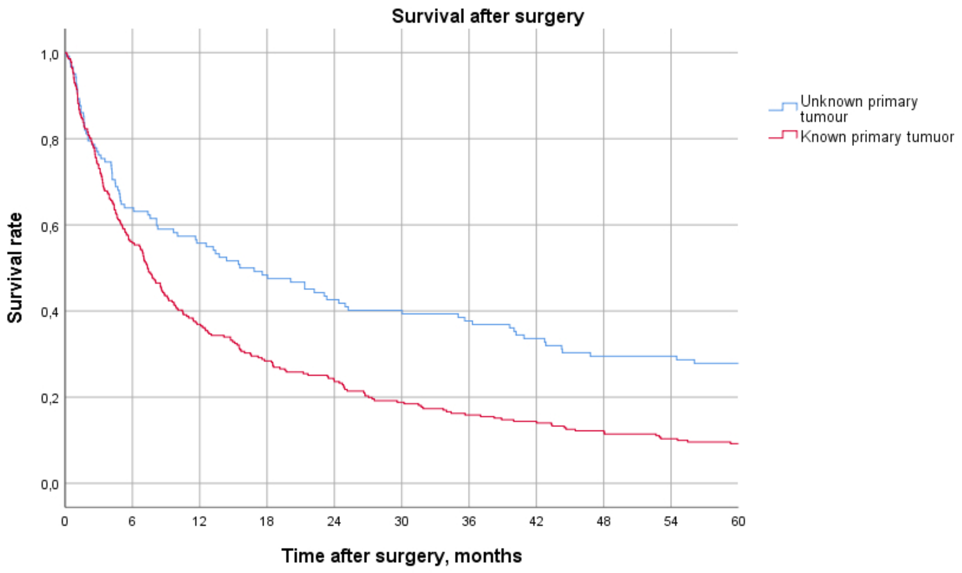

Kaplan Meier chart comparing survival in the UPT and KPT groups.

Reporting checklist for cohort study.

Based on the STROBE cohort guidelines.

Instructions to authors

Complete this checklist by entering the page numbers from your manuscript where readers will find each of the items listed below.

Your article may not currently address all the items on the checklist. Please modify your text to include the missing information. If you are certain that an item does not apply, please write "n/a" and provide a short explanation.

Upload your completed checklist as an extra file when you submit to a journal.

In your methods section, say that you used the STROBE cohortreporting guidelines, and cite them as:

von Elm E, Altman DG, Egger M, Pocock SJ, Gotzsche PC, Vandenbroucke JP. The Strengthening the Reporting of Observational Studies in Epidemiology (STROBE) Statement: guidelines for reporting observational studies.

For peer review only

		Reporting Item	Page Number
**Title and abstract**			
Title	#1a	Indicate the study’s design with a commonly used term in the title or the abstract	1
Abstract	#1b	Provide in the abstract an informative and balanced summary	2

		of what was done and what was found	
**Introduction**			
Background / rationale	#2	Explain the scientific background and rationale for the investigation being reported	4
Objectives	#3	State specific objectives, including any prespecified hypotheses	5
**Methods**			
Study design	#4	Present key elements of study design early in the paper	5
Setting	#5	Describe the setting, locations, and relevant dates, including periods of recruitment, exposure, follow-up, and data collection	6
Eligibility criteria	#6a	Give the eligibility criteria, and the sources and methods of selection of participants. Describe methods of follow-up.	6
Eligibility criteria	#6b	For matched studies, give matching criteria and number of exposed and unexposed	6
Variables	#7	Clearly define all outcomes, exposures, predictors, potential confounders, and effect modifiers. Give diagnostic criteria, if applicable	7
Data sources / measurement	#8	For each variable of interest give sources of data and details of methods of assessment (measurement). Describe comparability of assessment methods if there is more than one group. Give information separately for for exposed and unexposed groups if applicable.	8

For peer review only

Bias	#9	Describe any efforts to address potential sources of bias	8
Study size	#10	Explain how the study size was arrived at	8
Quantitative variables	#11	Explain how quantitative variables were handled in the analyses. If applicable, describe which groupings were chosen, and why	7
Statistical methods	#12a	Describe all statistical methods, including those used to control for confounding	

8

Statistical methods	#12b	Describe any methods used to examine subgroups and interactions	8
Statistical methods	#12c	Explain how missing data were addressed	12
Statistical methods	#12d	If applicable, explain how loss to follow-up was addressed	12
Statistical methods	#12e	Describe any sensitivity analyses	

12

Results

Participants	#13a	Report numbers of individuals at each stage of study—eg numbers potentially eligible, examined for eligibility, confirmed eligible, included in the study, completing follow-up, and analysed. Give information separately for for exposed and	9

For peer review only

		unexposed groups if applicable.	
Participants	#13b	Give reasons for non-participation at each stage	12
Participants	#13c	Consider use of a flow diagram	
8			
Descriptive data	#14a	Give characteristics of study participants (eg demographic, clinical, social) and information on exposures and potential confounders. Give information separately for exposed and unexposed groups if applicable.	6
Descriptive data	#14b	Indicate number of participants with missing data for each variable of interest	
11			
Descriptive data	#14c	Summarise follow-up time (eg, average and total amount)	
8			
Outcome data	#15	Report numbers of outcome events or summary measures over time. Give information separately for exposed and unexposed groups if applicable.	
7			
Main results	#16a	Give unadjusted estimates and, if applicable, confounder-adjusted estimates and their precision (eg, 95% confidence interval). Make clear which confounders were adjusted for and why they were included	9
Main results	#16b	Report category boundaries when continuous variables were	9

For peer review only

		categorized	
Main results	#16c	If relevant, consider translating estimates of relative risk into absolute risk for a meaningful time period	
9			
Other analyses	#17	Report other analyses done—eg analyses of subgroups and interactions, and sensitivity analyses	9
**Discussion**			
Key results	#18	Summarise key results with reference to study objectives	10
Limitations	#19	Discuss limitations of the study, taking into account sources of potential bias or imprecision. Discuss both direction and magnitude of any potential bias.	13
Interpretation	#20	Give a cautious overall interpretation considering objectives, limitations, multiplicity of analyses, results from similar studies, and other relevant evidence.	14
Generalisability	#21	Discuss the generalisability (external validity) of the study results	14
**Other Information**			
Funding	#22	Give the source of funding and the role of the funders for the present study and, if applicable, for the original study on which the present article is based	15

For peer review only

The STROBE checklist is distributed under the terms of the Creative Commons Attribution License CC-BY. This checklist was completed on 22. February 2021 using https://www.goodreports.org/, a tool made by the EQUATOR Network in collaboration with Penelope.ai

For peer review only
